# Locally advanced breast cancer arising in the axilla

**DOI:** 10.1093/jscr/rjac425

**Published:** 2022-09-19

**Authors:** Sasha Sapon-Cousineau, Dan Moldoveanu, Danielle Charpentier, Alain Gagnon, Érica Patocskai

**Affiliations:** Université de Montréal, Montréal, QC, Canada; McGill University, Montréal, QC, Canada; Centre Hospitalier de l'Université de Montréal, Montréal, QC, Canada; Centre Hospitalier de l'Université de Montréal, Montréal, QC, Canada; Centre Hospitalier de l'Université de Montréal, Montréal, QC, Canada

## Abstract

Locally advanced breast cancer arising from ectopic axillary breast tissue is an unusual presentation of this malignancy. The work-up and treatment approach pose some unique challenges. We present the case of a 37-year-old female presenting with a left axillary lesion with skin involvement. Radiological studies and biopsy demonstrated an underlying axillary mass compatible with a triple-positive invasive ductal carcinoma of the breast. Following neoadjuvant therapy, the patient underwent nipple-sparing mastectomy with wide local excision of the involved axillary skin and axillary lymph node dissection. Ectopic locally advanced breast cancer can be treated similarly to its orthotopic counterpart, favoring a neoadjuvant therapy approach followed by surgical excision. Special considerations include the local anatomy of the tumor, the extent of surgery and reconstructive options.

## INTRODUCTION

Ectopic breast tissue is present in about 0.6–6% of women [1]. It can be found along the mammary ridge, an embryonal formation extending from the axilla to the inguinal regions and the medial aspect of the thighs [2]. Abnormal regression of this formation results in leftover ectopic breast tissue [1]. Breast cancer can arise from this ectopic tissue, most commonly in the axilla (60–70% of cases) and is of similar type to orthotopic breast cancers [2]. Rarely, this may include locally advanced breast cancer (LABC) with skin involvement, which we present in this case report [3].

## CASE REPORT

A 37-year-old female was referred to our surgical oncology clinic for an enlarging left axillary mass. She had noticed a lesion 9 months prior to presentation, followed by erythema with skin thickening about 3 months later. Her family and personal history was unremarkable.

**Figure 1:**
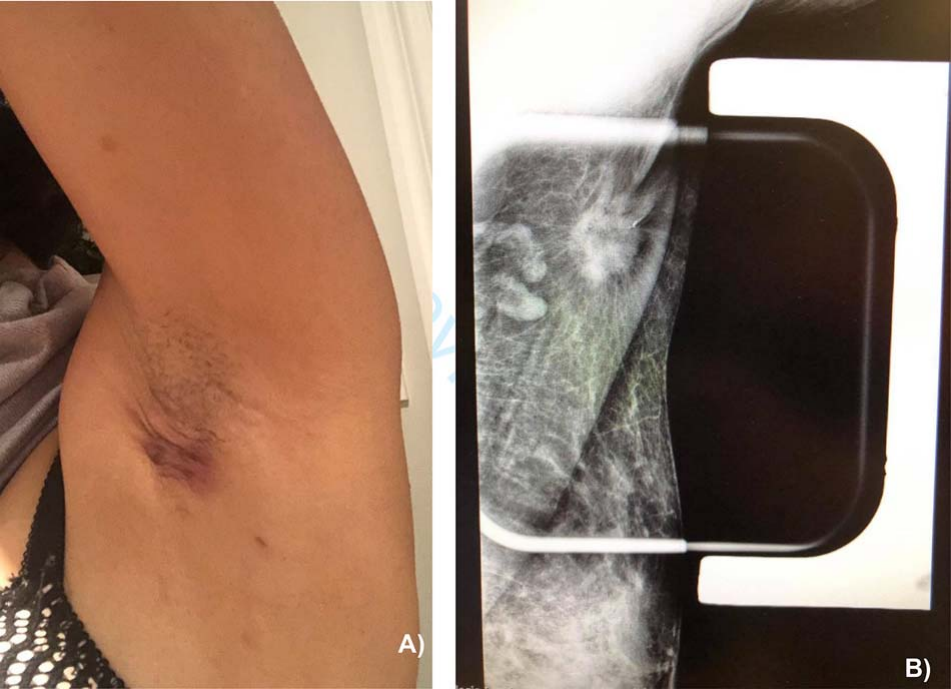
(**A**) Appearance of the left axillary lesion at presentation. (**B**) Magnified view on mammogram of the left axilla showing an irregular mass with extension to the skin in the context of an ectopic mammary gland.

**Figure 2:**
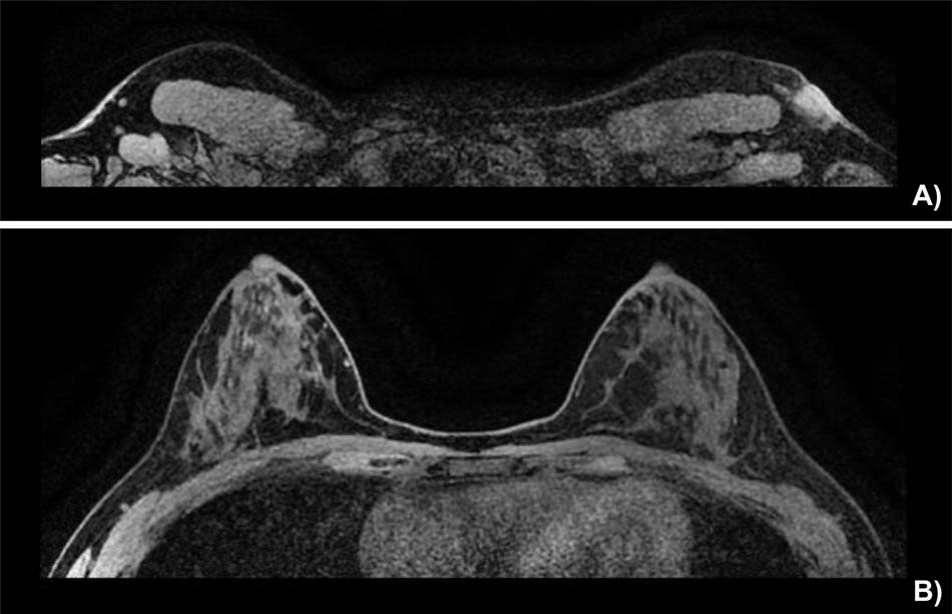
Breast MRI depicting (**A**) the known mass in a superficial axillary position with direct invasion of the overlying skin and dermal thickening, measured at 1.8 × 1.3 cm wide by 1.9 cm in height. The left axillary nodes appear more numerous and slightly enlarged but share no malignant features. (**B**) The left and right breast showing no sign of neoplasia.

On physical exam, the patient had an area of erythema measuring 4 × 3 cm in the left axilla, with palpable skin thickening and subcutaneous edema (Fig. 1A). An initial mammogram showed an irregular axillary lesion extending to the skin, arising in ectopic breast tissue (Fig. 1A). A subsequent ultrasound showed skin thickening and a spiculated mass measuring 8 mm within the axilla, without any abnormal axillary lymph nodes or breast lesions. Biopsy of the mass demonstrated invasive ductal carcinoma, which was estrogen receptor (ER) positive, progesterone receptor (PR) positive and HER-2 positive. The sample also contained benign mammary gland elements, supporting a tumor localization in ectopic axillary mammary tissue. A magnetic resonance imaging (MRI) study showed no suspicious breast lesions or axillary lymph nodes, and confirmed the known axillary mass with skin extension (Fig. 2). Complete staging by whole-body positron emission tomography (PET) scan and brain computed tomography (CT) scan showed no evidence of metastatic disease.

**Figure 3:**
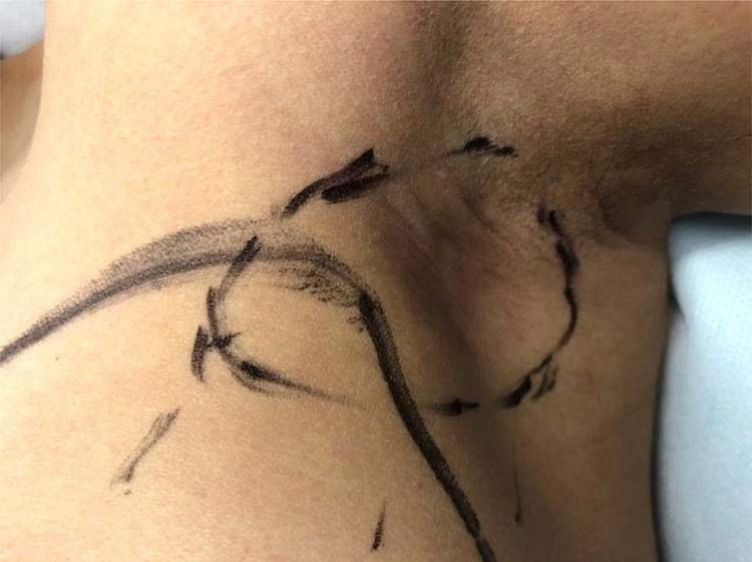
Pre-operative appearance of the left axillary lesion (circled in black) following neoadjuvant therapy.

Following discussion at our institution’s multidisciplinary tumor board, it was decided to proceed with neoadjuvant chemotherapy and targeted therapy. This consisted of dose dense cyclophosphamide/doxorubicin (AC), followed by paclitaxel with trastuzumab and pertuzumab. Upon completion of neoadjuvant therapy, the subcutaneous edema had regressed, and only slight erythema remained (Fig. 3). Ultrasound of the left breast and axilla after four cycles of neoadjuvant AC identified the lesion with persistent skin invasion. Skin thickening was measured at 3 mm and the mass at 12 mm. The total affected skin area was 2 cm in diameter. Genetic testing identified no known pathogenic mutations.

**Figure 4:**
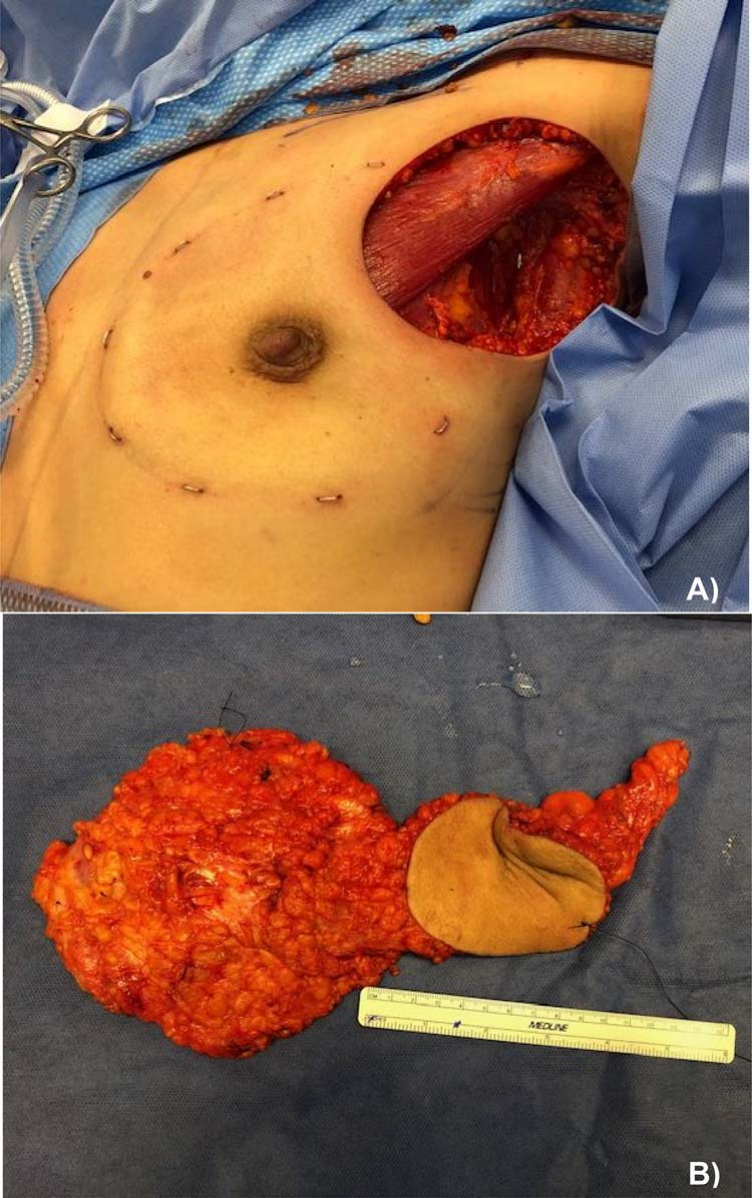
(**A**) Left nipple sparing mastectomy with axillary lymph node dissection and en-block skin excision and (**B**) pathological specimen containing from left to right: the total mastectomy, excised skin and axillary lymph node dissection.

Due to the extent of the disease and patient preference, a left nipple sparing mastectomy was performed, with en-block excision of the involved axillary skin and axillary lymph node dissection, followed by reconstruction with a thoracodorsal artery perforator latissimus dorsi flap. An expander was fitted under the pectoralis muscle and the cavity closed with an acellular dermal matrix (Fig. 4).

The final pathology report identified invasive ductal carcinoma in the dermis of the axillary skin as well as in the subjacent fibroadipose tissue with perineural invasion. Total radius of the neoplastic infiltration was 3.2 cm with negative resection margins. Two axillary nodes showed metastatic disease, with the largest tumor deposit measuring 0.3 cm. The left mastectomy specimen showed no malignancy. Receptor studies were hormone receptor positive and HER-2 positive.

Adjuvant systemic therapy and endocrine therapy were offered. Furthermore, the patient received standard radiotherapy to the left chest wall and axillary region.

## DISCUSSION

About 0.2–0.6% of all breast carcinomas arise in ectopic breast tissue [2]. These carcinomas tend to occur earlier and may have a higher rate of malignant degeneration, harboring benign lesions less frequently [1, 4]. A failure to recognize breast cancer in these unusual locations can lead to misdiagnosis and therapeutic delays.

We propose that the workup and treatment of ectopic LABC should follow the same principles as its orthotopic counterpart, with adjustments based on the loco-regional anatomy of the lesion and resectability. We take a detailed history and perform a physical examination of the breast, axillae and regional lymph nodes surrounding the lesion. A comprehensive workup including bilateral mammography and ultrasound should be performed. In cases of occult tumors presenting with isolated nodal disease or skin involvement, breast MRI may be warranted [5, 6]. Skin punch biopsies should be obtained to help differentiate LABC with skin involvement from inflammatory breast cancer [7]. A complete staging workup should be obtained, since occult metastases will be detected in 6–14% of patients with stage III disease [8]. Whole body PET/CT may be used for staging [8, 9]. Tumor ER/PR and HER-2 status guides hormonal and targeted therapies and helps define prognosis [10]. Genetic testing should be offered if indicated [11]. The therapeutic approach to LABC is determined by the molecular subtype of the tumor as well as the extent of the excision required to achieve negative margins [12]. Locally advanced triple-negative breast cancers, HER-2-positive cancers and hormone receptor-positive tumors that would require an extensive resection are best approached with neoadjuvant chemotherapy [13]. In the present case, neoadjuvant therapy was warranted by the large area of skin involvement and tumor subtype. Response to neoadjuvant treatment should be regularly evaluated. Radiological assessment should be carried out at the end of treatment or sooner depending on clinical response [13]. We determined response to treatment following four cycles of dose dense AC by ultrasound. Lesion size was found to be 12 mm, but comparison with initial ultrasound images was difficult due to the irregular nature of the mass. The global affected area was 2 cm – a partial response when compared with the initial measurement of 3 cm. Following neoadjuvant therapy, locoregional treatment may include mastectomy or breast conserving surgery with axillary staging or lymph node dissection [7, 14]. We excised the tumor to negative margins along with the contiguous breast, performing a left nipple sparing mastectomy and an axillary lymph node dissection. Given the ectopic location of the primary lesion, sentinel lymph node biopsy was deemed unreliable, as drainage patterns are less documented. Because of the partial clinical response, we delayed definitive reconstruction, allowing for adjuvant radiation therapy.

The overall prognosis of ectopic breast cancer is similar to its orthotopic counterpart, although diagnostic delays may result in larger tumors with a higher likelihood of lymph node involvement [2, 8]. An elevated degree of clinical suspicion and a sound understanding of the embryological origins of mammary tissue could prevent delays and misdiagnosis.

## DATA AVAILABILITY

All the relevant data were presented in the study.

## CONFLICT OF INTEREST STATEMENT

The author(s) declare that there is no conflict of interest regarding the publication of this article.

## FUNDING

No funding was received for the publication of this article.
